# Live-Cell Visualization of Intracellular Interaction between a Nuclear Migration Protein (hNUDC) and the Thrombopoietin Receptor (Mpl)

**DOI:** 10.1371/journal.pone.0051849

**Published:** 2012-12-17

**Authors:** Yuan-Bin Zheng, Ying-Ying Xiao, Peng Tan, Qing Zhang, Peilin Xu

**Affiliations:** The Key Laboratory of Gene Engineering of Education Ministry, Zhongshan University, Guangzhou, The People’s Republic of China; University of Melbourne, Australia

## Abstract

We previously demonstrated that endogenous hNUDC and Mpl co-localized in the perinuclear and cytoplasmic regions of megakaryocyte cells by indirect immunofluorescence. We further reported that hNUDC accumulated in the Golgi when NIH 3T3 cells were transfected with an hNUDC expression vector alone. However, co-transfection with hNUDC and Mpl expression vectors caused both proteins to co-localize predominantly in the cytosol. These observations led us to hypothesize that a complex containing hNUDC and Mpl may alter hNUDC subcellular location and induce its secretion. In the present study, we test this hypothesis by employing bimolecular fluorescence complementation (BiFC) to detect and visualize the complex formation of hNUDC/Mpl in living cells. We further examined in detail the subcellular locations of the hNUDC/Mpl complex by co-transfection of BiFC chimeras with known subcellular markers. The distribution of hNUDC/Mpl in the endoplasmic reticulum (ER), Golgi and cell surface was determined. Furthermore, the N-terminal 159 amino acids of hNUDC, but not C-terminal half, bound to Mpl *in vivo* and exhibited a similar localization pattern to that of full-length hNUDC in Cos-1 cells. Adenovirus-mediated overexpression of hNUDC or its N-terminal 159 residues in a human megakaryocyte cell line (Dami) resulted in increased levels of hNUDC or hNUDC(1-159) secretion. In contrast, depletion of Mpl by transfecting Dami cells with adenovirus bearing Mpl-targeting siRNA significantly blocked hNUDC secretion. Thus, we provide the first evidence that the N-terminal region of hNUDC contains all of the necessary information to complex with Mpl and traffic through the secretory pathway.

## Introduction

Human NUDC (hNUDC) was initially identified and cloned as a nuclear migration protein based on the fact that its C-terminal 96 amino acids are 68% identical to the filamentous fungus *Aspergillus nidulans* NUDC [1], [2]. The high amino acid homology at the C-terminal residues between human and fungal NUDC suggest that such regions might be preserved during evolution because it carries out an essential cellular function in fungi and humans [3], [4]. Like fungal NUDC, many of the earlier studies have emphasized that hNUDC is an intracellular microtubule-associated protein involved in cell mitosis and cytokinesis [5]–[7]. However, upon sequence alignment of hNUDC with mouse, rat or other higher eukaryotic NUDC, it is evident that these mammalian homologues typically possess additional N-terminal extensions not present in fungal NUDC. Therefore it is posited that the functional roles of the hNUDC N-terminus have shifted during their evolution [3], [4]. However, until now the specific roles of this N-terminal extension have remained elusive.

The widespread tissue distribution of NUDC mRNA has been previously mapped in humans [8]. The degree of hNUDC mRNA expression is particularly elevated in clinical bone marrow isolates from patients with acute lymphoblastic or acute myelogenous leukemia [8], which suggests that hNUDC may have evolved to convey additional functions in hematopoietic cells [8], [9]. Recently, we have shown that hNUDC acts as a second natural ligand for thrombopoietin (TPO) receptor (Mpl), and it produces similar biological effects as TPO when it is present in the extracellular medium [10], [11]. We have also confirmed the specificity of the interaction between hNUDC and Mpl and identified the binding domains in each molecule by a yeast two-hybrid, GST Pull-down, co-immunoprecipitation and phage-display studies [12]–[14]. In addition, we have also demonstrated that the co-expression of hNUDC and Mpl effectively elevates hNUDC secretion in NIH 3T3 cells, leading us to propose a mechanism of Mpl-dependent secretion of hNUDC [13].

In this study, we aimed at further probing these interactions using a recently developed Venus-based bimolecular fluorescence complementation (BiFC) system [15]–[18] in conjunction with flow cytometry and fluorescence resonance energy transfer (FRET) to examine hNUDC/Mpl interaction in living cells. We demonstrated that hNUDC interacts with Mpl in an N-terminus-dependent manner. In addition, the possible associations of BiFC complexes with ER, Golgi and cell membrane were examined. Moreover, we used an adenovirus carrying either full hNUDC or a C-terminal truncated version into Dami cells and assayed protein accumulation in the conditioned medium. We have also tested for inhibition of secretion of hNUDC or its N-terminal half by employing knockdown of Mpl under the same conditions. The results presented here advance our understanding of how the cellular release of functional hNUDC is mediated by the intracellular interaction of hNUDC and Mpl.

## Materials and Methods

### Construction of Chimeras for BiFC Analysis

Cloning vectors pBiFC-VN173 and pBiFC-VC155, which express Flag-tagged-N-terminal 1-173 aa of Venus (VN) and hemagglutinin (HA)-tagged-C-terminal 155–238 aa of Venus (VC) fusion proteins, respectively, were kindly provided by Dr Chang-Deng Hu (Purdue University, West Lafayette, IN). The cDNA fragments encoding the full-length (1-331), N-terminal half (1-159) and C-terminal half (160-331) of hNUDC were amplified from phNUDC-DsRed [13] using primers containing the appropriate restriction sites. The resulting PCR fragments were cloned in-frame into the corresponding restriction sites between HA and VC in pBiFC-VC155. A cDNA fragment encoding full-length Mpl was amplified by PCR from pMpl-EGFP [13] using primers containing the appropriate restriction sites and then inserted in-frame into the corresponding restriction sites between Flag and VN in pBiFC-VN173. The sequence of each construct used in this study was verified by restriction mapping and nucleotide sequence analysis.

### BiFC Analysis by Confocal Microscopy

Cos-1 cells purchased from American Type Culture Collection (ATCC) were cultured in Dulbecco’s modified Eagle’s medium (DMEM) with 10% fetal bovine serum (FBS), 10 units/ml penicillin, and 100 µg/ml streptomycin. For BiFC visualization, Cos-1 cells were cultured on coverslips in 6-well dishes and transiently transfected with VN and VC expression vectors using Lipofectamine 2000 reagent (Invitrogen, Carlsbad, CA) according to the provider’s instructions. After 12 h incubation at 37°C, the coverslips were placed directly on a drop of culture media on a glass slide and examined with a Leica SP5 Spectral Confocal Laser Scanning Microscope (Leica Microsystems CMS GmbH, Germany) controlled by Leica LAS AF software. Fluorescence resulting from the interaction of the VN and VC proteins was detected by excitation at 515 nm and emission at 528 nm. The level of expression of each fusion protein was quantified by Western blotting using antibodies directed against the Flag or HA epitope (Santa Cruz Biotechnology, Santa Cruz, CA).

### BiFC Analysis by Flow Cytometry

For flow cytometry, cells were grown in 6-well plates and transfected with the YN and YC expression vectors. After 12 h incubation at 37°C, cells were gently trypsinized and collected into 15 ml Falcon tubes. Cells were then pelleted by centrifugation, washed twice with ice-cold phosphate-buffered saline (PBS), and resuspended in 100 µl of the same solution. Fluorescence-activated cell sorting (FACS) analysis was performed in a FACSCalibur flow cytometer (BD Biosciences, San Jose, Ca) with CellQuest software using an excitation wavelength of 488 nm. Suspensions of 1×10^4^ cells were assayed for the percentage of Venus-positive cells with FACSVantage Software. Experiments were performed at least three times. The data (expressed as the percentage of Venus-positive cells) shown in the figures were taken from representative experiments. Statistical significance was determined using one-way ANOVA. Student’s t tests with *p*<0.05 were considered statistically significant.

### Acceptor Photobleaching FRET Assay

Sequences for full-length of hNUDC, hNUDC(1-159) and hNUDC(160-331) were amplified by PCR from pHA-hNUDC-VC and inserted upstream and in frame with the Cyan fluorescent protein (CFP) in pECFP-N1(Clontech, Mountain View, CA), generating phNUDC-CFP, phNUDC(1-159)-CFP and phNUDC(160-331)-CFP, respectively. Sequence for Mpl was amplified by PCR from pFlag-Mpl-VN and inserted upstream and in frame with the yellow fluorescent protein (YFP) in pEYFP-N1(Clontech, Mountain View, CA), generating pMpl-YFP. Plasmids phNUDC-CFP, phNUDC(1-159)-CFP and phNUDC(160-331)-CFP were co-transfected with pMpl-YFP into Cos-1 cells respectively. After 24 h transfection, cells were washed twice with PBS and fixed using 4% paraformaldehyde in PBS. Photo-bleaching was performed by repeated scanning a ROI (region of interest) using the 514 nm at maximum intensity. An excitation wavelength of 458 nm and an emission wavelength of 470–500 nm were used for CFP, and an excitation wavelength of 514 nm and an emission wavelength of 515–545 nm were used for YFP [19]. The FRET energy transfer efficiency (E_f_) was calculated as FRET_eff_ = (I_post_ − I_pre_)/I_post_ where I_pre_ and I_post_ are the total fluorescence of the ROI before and after bleaching.

### Construction of Vectors for Expression of the DsRed-monomer Fluorescent Protein (mCherry)-tagged Subcellular Localization Markers

For the generation of a vector expressing Golgi-localized protein (pmCherry-Golgi), total RNA from Hela cells was isolated using TRIzol® Reagent (TaKaRa Biotechnology, Dalian, China) according to the manufacturer’s instructions. The first-strand cDNA was synthesized using a First-Strand cDNA Synthesis Kit (TaKaRa Biotechnology, Dalian, China) according to the manufacturer’s instructions. The sequence encoding the N-terminal 1–81 aa of human β-1-4-galactosyltransferase, which is traditionally used as a Golgi marker [20], was amplified from HeLa cDNA using forward primer 5′-GATCAAGCTTATGTCCGGCCCCACCATG-3′ containing a *Hind*III site (underlined) and reverse primer 5′-GATCGTCGACGCCCAGCCAGTCTGGGTC-3′ containing a *Sal*I site (underlined). The PCR product was inserted upstream and in-frame with the mCherry in pDsRed-monomer-N1 (Clontech, Mountain View, CA).

To create expression plasmid encoding an ER marker (pmCherry-ER), the sequences coding for Lys-Asp-Glu-Leu (KDEL) [21] were fused to the C terminus of mCherry by PCR-driven amplification using forward primer 5′-CGGGATCCAATGGACAACACCGAGG-3′ containing an *Eco*RI site (underlined) and reverse primer 5′-CCGCTCGAGCTTA***CAGCTCGTCCTT***CTGGGAGCCGGAGTGGCG-3′ containing a *Xho*I (underlined; bold and italic indicate KDEL). A mCherry-KDFL PCR fragment was inserted into pSecTag2/HygroA (Invitrogen, Carlsbad, CA).

For generation of the cell membrane marker (pmCherry-MB), the sequence encoding mCherry was amplified by PCR using sense primer 5′-TCCCCGCGGCATGGACAACACC-3′ containing *Sac*II (underlined) and antisense primer 5′-ACGCGTCGACCTGGGAGCCGGAGTG-3′ containing *Sal*I (underlined), and inserted upstream and fused in-frame at the N-terminus to the murine Ig κ-chain leader sequence in pDisplay (Invitrogen, Carlsbad, CA).

### Subcellular Localization of BiFC Interaction

For analysis of subcellular localization, Cos-1 cells were triple transfected with an individual mCherry fused marker together with the BiFC chimeric plasmids on coverslips in six-well plates. After 12 h incubation at 37°C, Venus fluorescence was excited at 514 nm and detected at 527 nm. DsRed-monomer was excited at 556 nm and detected at 586 nm. DAPI was employed as a nuclear stain.

### Adenovirus Construction for Expression of hNUDC

The cDNA of full-length hNUDC and both N- and C-terminal truncated versions of hNUDC were amplified by PCR using a set of primers to create a *Sal*I site at the 5′ end and a *Xba*I site at the 3′ end; these PCR fragments were then inserted into the same restriction sites of the pAdTrack-CMV shuttle vector (Fisher Scientific, Shanghai, China) which contains the green fluorescent protein (GFP) reporter gene for monitoring the efficiency of adenovirus infection. The pAdTrack-CMV clones were digested with *Pme*I and transformed into BJ5183 cells containing adenoviral backbone vector pAdEasy-1 for recombinant generation of the adenoviruses carrying hNUDC, hNUDC(1-159) or hNUDC(159-331). Kanamycin-resistant colonies were selected and analyzed by restriction digestion. The plasmid DNA from correct clones was linearized with *Pac*I and transfected into HEK293 cells for the generation of recombinant adenoviruses, designated Ad-hNUDC, Ad-hNUDC(1-159) and Ad-hNUDC(160-331), respectively. Recombinants adenoviruses were plaque-purified and amplified. Aliquots of the virus stocks were frozen in liquid N_2_ and stored at -80°C. Generally, viral titers of the stocks varied from 1 to 5×10^10^ plaque-forming units (PFUs)/ml.

### Adenovirus Construction for the Expression of Mpl-targeting Small Hairpin RNA (shRNA)

The Mpl small interfering RNA (siRNA) primers used were as described previously [22]. We used the adenoviral pShuttle-CMV, which was reengineered to express GFP, to create an adenovirus vector expressing shRNA targeting Mpl (Ad-shRNA-Mpl) or a nonspecific sequence (Ad-shRNA-scramble). Ad-shRNA-Mpl or Ad-shRNA-scramble were used to infect HEK293 cells, and subsequently purified and tittered as described above.

### Adenoviral Transfection of Dami Cells

Dami cells purchased from ATCC were grown in Iscove’s modified Dulbecco’s medium (IMDM) supplemented with 10% FBS, and 1% penicillin/streptomycin/glutamate. Transfection of Dami cells was accomplished by exposing cells to recombinant viruses at a concentration of 1×10^9^ PFU/ml in serum-containing medium. When GFP expression was greater than 90%, cells were washed twice with IMDM by addition of serum-free medium (IMDM medium plus 100 U/ml penicillin, 100 *µ*g/ml streptomycin and 1% Nutridoma).

### Western Blotting to Assay Protein Secretion

Dami cells were cultured and transfected with Ad-hNUDC, Ad-hNUDC(1-159) or Ad-hNUDC(160-331) as described above. Twenty-four hours after transfection, cells maintained in IMDM were serum-starved overnight. Cells were rapidly washed with ice-cold PBS and incubated in serum-free conditioned media. To confirm that >98% of cells were alive, an aliquot of cells was checked for cell death under a microscope after staining with Trypan Blue. The supernatants of Ad-hNUDC-, Ad-hNUDC(1-159)- or Ad-hNUDC(160-331)-transfected cultures were harvested after 0, 6, 12, 24, 36 and 48 h. For Mpl knockdown, Dami cells were co-transfected with Ad-shRNA-Mpl and Ad-hNUDC or Ad-shRNA-Mpl and Ad-hNUDC(1-159). Co-transfection of a non-targeting siRNA with either Ad-hNUDC or Ad-hNUDC(1-159) was used as the control. At different time points, conditioned media were electroblotted to polyvinylidene fluoride membranes using a semidry electrophoretic transfer unit (Amersham Biosciences, NJ). The membrane was blocked in a mixture of TBS containing 50 mM TrisHCl pH7.4, 150 mM NaCl, 0.1% Tween-20 and 5% nonfat milk. After washing, the membrane was incubated with anti-hNUDC antibodies diluted in the blocking mixture. Immunoreactive bands were visualized with a NBT/BCIP Kit (Roche, Indianapolis, IN) using alkaline phosphatase (AP)-labeled secondary antibodies.

## Results

### BiFC Complex Formation of hNUDC and Mpl in Living Cells

**Figure 1:**
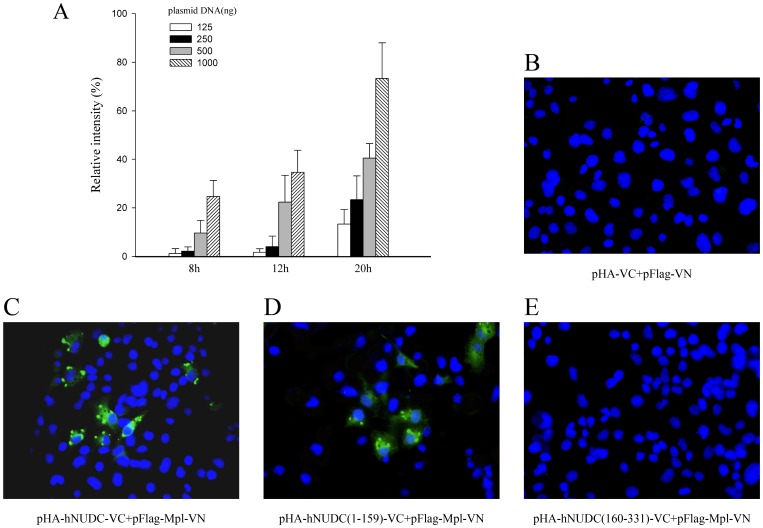
The protein-protein interactions of BIFC constructs in living cells. (**A**) BiFC detected by flow cytometry of Cos-1 cells transfected with empty vectors, pBiFC-VN173 and pBiFC-VC155 by varying amounts of DNAs (125–1000 ng) at each time point as indicated. (**B**) Confocal image of Cos-1 cells gave no detectable Venus fluorescence when 250 ng of DNA for control vectors were used after 12h transfection. (**C–E**) Fluorescence images of Cos-1 cells expressing the VC and VN pairs indicated in each panel under the same conditions. The strong BIFC signal was monitored by confocal microscopy in hNUDC-VC/Mpl-VN- or hNUDC(1-159)-VC/Mpl-VN-transfected cells, but no fluorescence was seen in cells co-transfected with hNUDC(160-331)-VC and Mpl-VN. The cells were stained with DAPI to indicate the position of cell nucleus (Original magnification, 40x).

The goal of this work was to further explore the interaction of hNUDC with Mpl using a BiFC assay that allows direct visualization of protein complex formation in living cells. For this purpose, we generated expression vectors that fused full-length hNUDC in-frame to the C-terminal half of Venus (designated pHA-hNUDC-VC) and full-length Mpl with the N-terminal half of Venus (designated pFlag-Mpl-VN). To avoid nonspecific interaction occur between the YN or YC fragments, we initially determined a concentration-and time-dependent analysis of VC and VN fluorescence by testing cells with a range of empty vector DNA (125–1000 ng) at 8, 12 and 20 h (Fig. 1A). Flow cytometry analysis showed that co-transfection of Cos-1 cells with an equal amount of empty vectors at 125 or 250 ng resulted in very low fluorescence from 8 to 12 h. However, fluorescence intensity was increased when 500 or 1000 ng of DNA was transfected under the same conditions (Fig. 1A). As a result of these initial studies, we therefore followed a protocol by using 250 ng DNA for each BiFC construct and assayed 12 h after transfection. As expected, co-transfection of Cos-1 with the empty vectors did not show any fluorescence (Fig. 1B). In contrast, cells co-expressing HA-hNUDC-VC and Flag-Mpl-VN exhibited bright granular or punctuate structures (Fig. 1C). Because hNUDC contains an N-terminal extension region and a C-terminal conserved domain, it prompted us to analyze which is required for Mp1 binding *in vivo.* We next prepared N-terminal and C-terminal deletion mutants by fusing hNUDC residues 1-159 and 160-331 of hNUDC in-frame to the C-terminal halves of Venus, designated pHA-hNUDC(1-159)-VC and pHA-hNUDC(160-331)-VC, respectively. Cells transfected with HA-hNUDC(1-159)-VC and Flag-Mpl-VN exhibited a level of Venus fluorescence that was comparable to that of HA-hNUDC-VC and Flag-Mpl-VN (Fig. 1D). However, no fluorescence was observed when HA-hNUDC(160-331)-VC was co-expressed with Flag-Mpl-VN (Fig. 1E). These results indicate that hNUDC can specifically interact with Mpl through its N-terminal half *in vivo*.

### Flow Cytometry Analysis of BiFC

**Figure 2:**
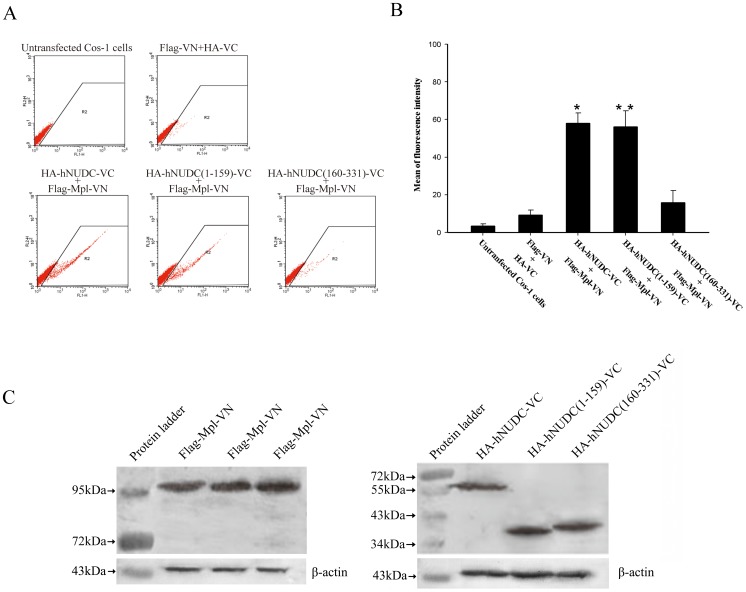
The BiFC was quantified by flow cytometric analysis and Western blot. Cos-1 cells were transfected with pairs of BiFC constructs or with control constructs as indicated for 12 h. For each combination, the mean fluorescent intensity of YFP from 1×10^4^ cells was determined. (**A**) Dot plots showing the R2 gate delimiting the area of BIFC signals. (**B**) The relative levels of the specific fluorescence signals are shown as a histogram. Data are representative of at least 4 independent experiments. *, p<0.05; **, p<0.01 compared to untransfected Cos-1 cells. (**C**) Examination of expression levels of BiFC constructs by Western blot. Cos-1 cells co-expressing the indicated BiFC constructs were lysed and incubated with anti-HA or Flag antibodies. Locations of molecular weight markers are indicated.

Flow cytometry allows for the quantification of the relative efficiency of BiFC complex formation by the determining of the number of Venus-expressing cells relative to the total number of cells. As shown in Fig. 2A, the percentage of Cos-1 cells with fluorescence of that expressing hNUDC-VC/Mpl-VN or hNUDC(1-159)-VC/Mpl-VN emitted fluorescence signal far in excess of the fluorescence observed with untransfected cells or control vector transfected cells. The increases of BiFC fluorescence observed with hNUDC-VC/Mpl-VN and hNUDC(1-159)-VC/Mpl-VN co-expressions were statistically significant, with *P* values of <0.05 and 0.001, respectively, as determined by two-tailed *t* tests (Fig. 2B). Although the percentage of cells showing Venus fluorescence was greater with hNUDC(160-331)-VC/Mpl-VN co-expression than with the control vectors, this was not statistically significant by ANOVA (Fig. 2B). Overall, there was a very good correlation between the results of flow cytometry analysis and fluorescence microscopy. We next performed immunoblotting in parallel to assay protein expression with either a monoclonal anti-HA or anti-Flag antibody. As demonstrated in Fig. 2C, transfection with fusion constructs resulted in near-equal levels of protein expression, with bands of the expected molecular weight. Equal amounts of proteins were loaded as indicated by probing for the loading control β-actin (Fig. 2C).

### Interaction of hNUDC and Mpl is Confirmed by Acceptor Photobleaching FRET

**Figure 3:**
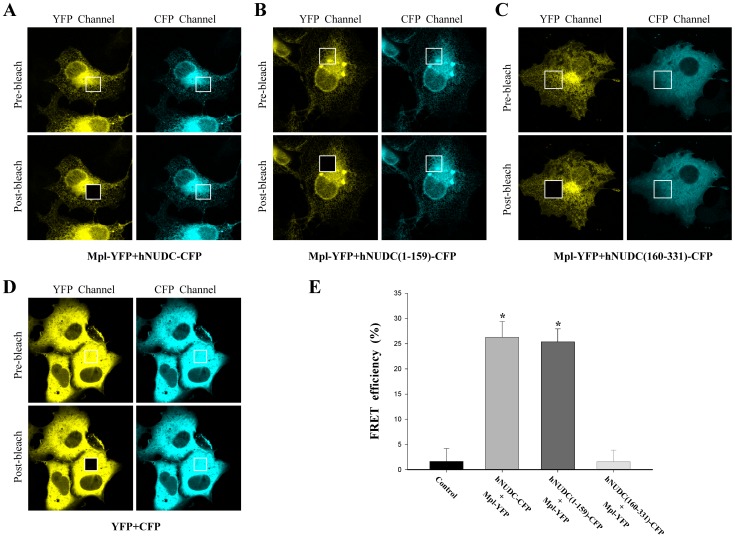
Cell images from standard acceptor photobleaching FRET. (**A–C**) Cos-1 cells transfected with plasmids encoding the CFP-YFP fusion proteins as indicated. (**D**) Representative cells transfected with empty vectors as a negative control for FRET. The white boxes indicated CFP and YFP images before and after photobleaching by a high intensity argon laser light. Five ROIs were analyzed for each cell and at least 5 cells were quantified from three different experiments. (**E**) FRET efficiencies calculated with Leica LAS AF software during YFP photobleaching. All data are represented as mean±S.D. *, P<0.05 compared to untransfected Cos-1 cells.

To validate the results of BiFC analysis, we further carried out a FRET assay by co-transfection of pMpl-YFP with phNUDC-CFP, phNUDC(1-159)-CFP and phNUDC(160-331)-CFP into Cos-1 cell respectively. At 24 h after transfection, energy transfer between CFP and YFP was detected by acceptor photobleaching at a specific location in the cell marked by the boxes (Fig. 3, A-D). As shown in Fig. 3E, donor CFP fluorescence increased 25% and 24% upon bleaching (post-bleach) in the phNUDC-CFP/pMpl-YFP and phNUDC(1-159)/pMpl-YFP samples. As expected, FRET efficiency of sample co-transfected with phNUDC(160-331)-CFP and Mpl-YFP showed no increase in the donor CFP fluorescence intensity in comparison to control, which is indicative of the lack of interactions between the two proteins (Fig. 3E).

### Subcellular Location of BiFC Constructs

**Figure 4:**
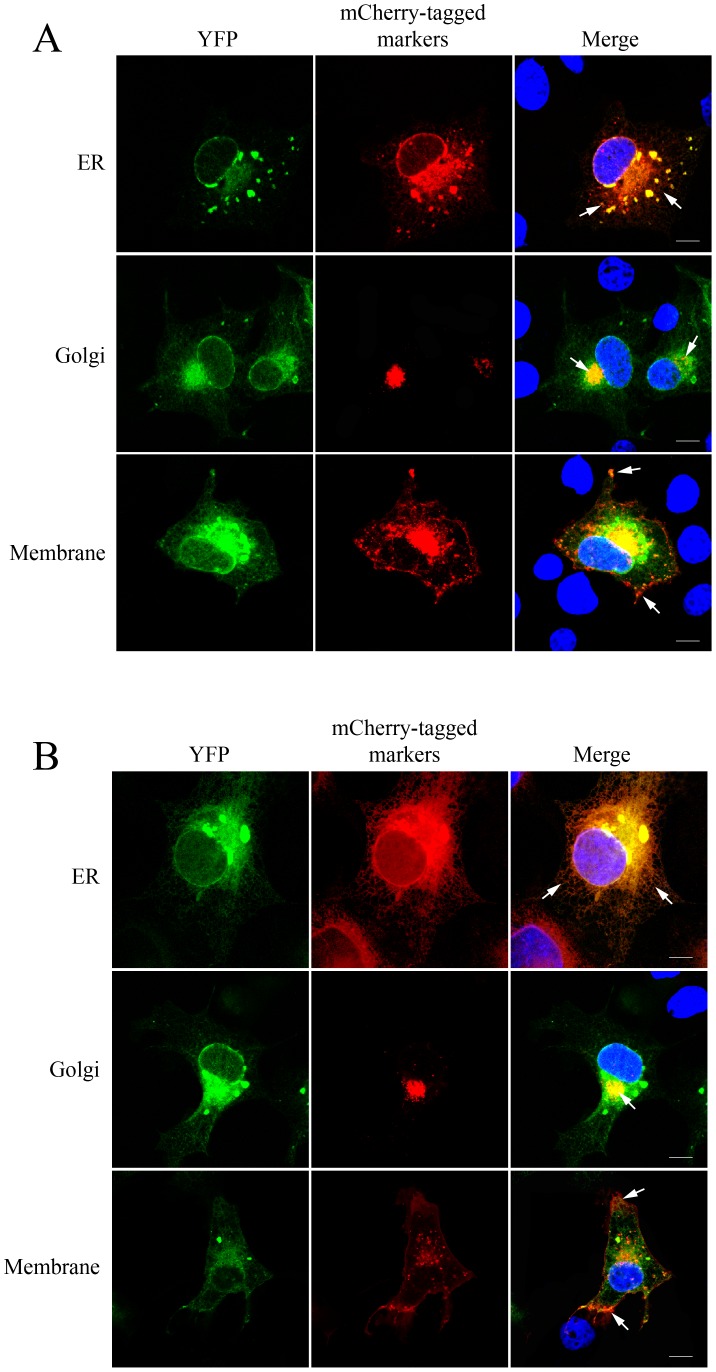
Subcellular localization of BiFC constructs. (**A**) Representative BiFC distribution of hNUDC-VC/Mpl-VN is shown. (**B**) Representative BiFC distribution of hNUDC(1-159)-VC/Mpl-VN is shown. BiFC constructs were co-transfected with the indicated subcellular markers. Fluorescent images shown were captured 12 h after transfection. Merged images show good co-localization of hNUDC/Mpl or hNUDC(1-159)/Mpl with the ER, Golgi and cell membrane. The cells were stained with DAPI to indicate the position of cell nucleus. Arrows, regions of co-localization; scale bar, 5 µm.

To facilitate immunofluorescence detection of the subcellular locations of the hNUDC/Mpl complex, we developed a series of plasmids encoding mCherry-tagged Golgi, ER and cell membrane markers. Fluorescence microscopy of pmCherry-ER-, pmCherry-Golgi- or pmCherry-MB-transfected cells indicated successful ER, Golgi and cell surface expressions (Fig. 4A). An individual marker plasmid together with pHA-hNUDC-VC/pFlag-Mpl-VN was then triple co-transfected into Cos-1 cells. As expected, a portion of the fluorescent hNUDC-VC/Mpl-VN complex overlapped with the ER marker, which was seen throughout the cytoplasm as punctate and reticular structures. In some cells, the intense fluorescence signal of Venus-tagged hNUDC-VC/Mpl-VN complex in the perinuclear region was found partially overlapping with β-1,4-GT, which demarcates the Golgi region (Fig. 4A). We observed a faint fluorescence diffusely distributed throughout the cell, most likely caused by a cytosolic form of hNUDC-VC/Mpl-VN attached to the cell membrane marker (Fig. 4A). As seen in Fig. 4B, co-expression of hNUDC(1-159)-VC coupled with Mpl-VN revealed identical subcellular expression patterns to that of hNUDC-VC/Mpl-VN. These results are consistent with the hypothesis that the trafficking of hNUDC/Mpl complex out of the ER and into the cell membrane is primarily mediated by the N-terminus of hNUDC.

### Secretion of hNUDC from Dami Cells

**Figure 5:**
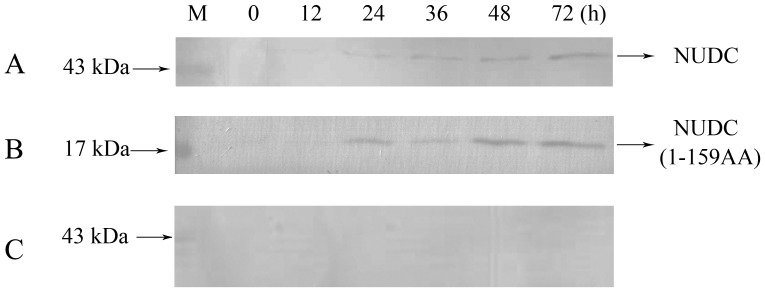
Western blot analysis of hNUDC domains in the medium of cultured Dami cells. (**A**) Dami cells were transfected with Ad-hNUDC. (**B**) Dami cells were transfected with Ad-hNUDC(1-159). (**C**) Dami cells were transfected with Ad-hNUDC(160-331). Secreted hNUDC, hNUDC(1-159) or hNUDC(160-331) in the culture medium was assayed by immunoblot using an anti-hNUDC antibody at each time point indicated.

The fact that the cellular localization of the hNUDC/Mpl BiFC construct was identical to that of endogenous hNUDC and Mpl co-localization in megakaryocyte cells [10] leads to the possibility that such intracellular interactions had a similar stimulatory effect on the secretion of hNUDC in different cell types. We next used a megakaryocyte cell line, which has the advantage of endogenous Mpl expression, for further confirmation of whether overexpression of hNUDC or hNUDC(1-159) enhances the accumulation of hNUDC in the medium of cultured Dami cells. Dami cells transfected with 10^9^ PFU/ml of Ad-NUDC, Ad-hNUDC(1-159), or Ad-hNUDC(160-331) were incubated in serum-free media over a period of 0–72 h, and the amount of secreted hNUDC in conditioned media was analyzed by Western blotting. Notably, secretion of hNUDC or hNUDC(1-159) into the medium remained at a relatively lower level at 12 h, but increased 24 to 72 hours after transfection (Fig. 5). We assayed the molecular weight of hNUDC based on electrophoretic mobility, and found that the predominant forms of hNUDC and hNUDC(1-159) migrated at ∼45kDa and ∼19kDa, respectively (Fig. 5). In contrast, no signal was seen in the medium of Dami cells transfected with Ad-hNUDC(160-331) (Fig. 5). The secretion of hNUDC was not due to cell damage because minimal amounts of dead cells were detected in the incubation medium during these experiments (data not shown).

### Knockdown of Mpl Expression Inhibits hNUDC Secretion in Dami Cells

**Figure 6:**
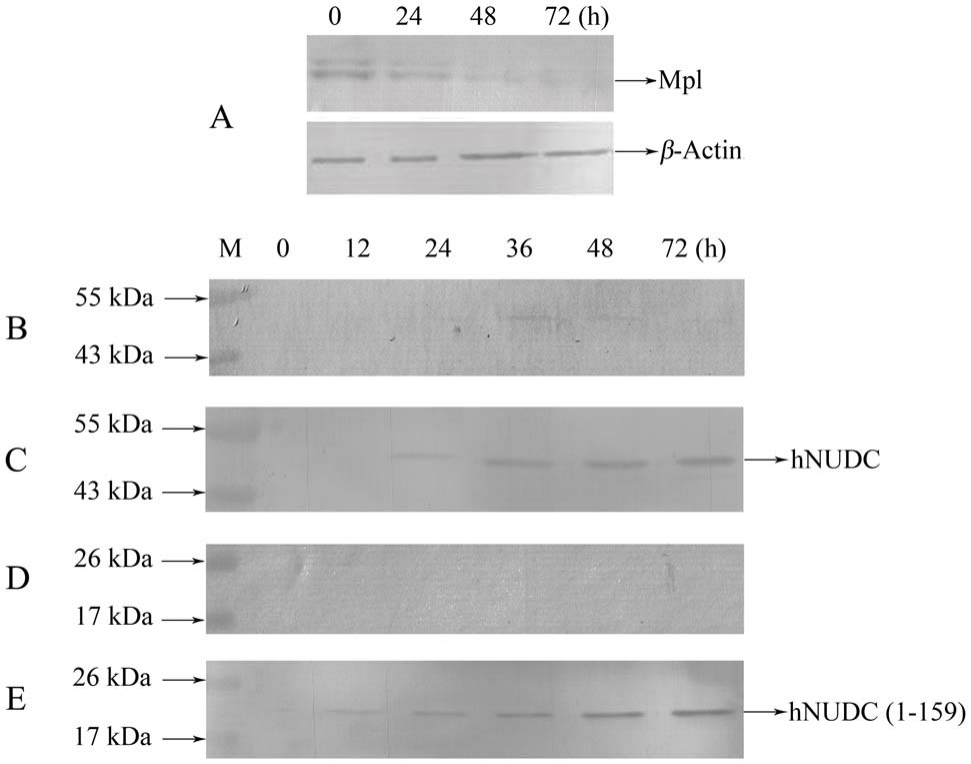
Western blot analysis of hNUDC domains in the medium of cultured from Mpl knockdown Dami. (**A**) Effects of siRNA-mediated Mpl knockdown. Dami cells were transfected with Ad-ShRNA-Mpl. Total cell proteins were prepared at the indicated times, and the effects of Ad-ShRNA-Mpl transfection on Mpl expression levels were evaluated by immunoblotting using an anti-Mpl antibody. Determination of the protein levels of β-Actin (bottom) was used as a control to ensure equal protein loading. (**B**) Time course of hNUDC released into culture medium after Dami cells were co-transfected with Ad-hNUDC and Ad-ShRNA-Mpl. (**C**) Comparison of hNUDC released into culture medium in cells transfected with Ad-hNUDC and control Ad-shRNA-scramble. (**D**) Time course of hNUDC released into culture medium after Dami cells were co-transfected with Ad-hNUDC(1-159) and Ad-ShRNA-Mpl. (**E**) Comparison of hNUDC(1-159) release into culture medium in Dami cells transfected with Ad-hNUDC(1-159) and control Ad-shRNA-scramble. Proteins released into the culture medium were analyzed by immunoblot using an anti-hNUDC antibody at each time point as indicated. Results are from 3–5 independent experiments.

If Mpl regulates hNUDC release via direct interactions with each other, knockdown of Mpl expression should exert an effect on the secretion of hNUDC. We next investigated the involvement of Mpl in the secretion of hNUDC by producing an adenoviral vector that would express a shRNA targeting Mpl in Dami cells [22]. To ensure efficient siRNA knockdown of Mpl, we first examined the expression levels of Mpl in Dami cells lysates. Immunoblot analysis, as shown in Fig. 6, indicated that Ad-shRNA-Mpl progressively reduced Mpl expression and generated significant knockdown at 24–72 h. No change in the expression level of β-actin was observed under the same conditions. Next, Dami cells were co-transfected with an equivalent amount of Ad-shRNA-Mpl and either Ad-hNUDC or Ad-hNUDC(1-159) and incubated in serum-free medium for various periods up to 72 h. As shown in Fig. 6B and 6D, knockdown of Mpl completely inhibited hNUDC and hNUDC(1-159) secretion in the conditioned media. A parallel experiment in which cells were co-transfected with control Ad-shRNA-scramble and either hNUDC or hNUDC(1-159) showed no changes in the secretion of hNUDC or hNUDC(1-159) (Fig. 6C and 6E).

## Discussion

Various techniques, including yeast two-hybrid, co-immunoprecipitation, phage-display and GST pull-down, have been previously utilized to detect the interaction between hNUDC and Mpl [12]–[14]. However, the most of these methods require removal of the proteins from their native environment, which generally prevents determination of the exact subcellular localization of the interacting proteins. The BiFC method overcomes these limitations and enables a detailed analysis of protein complex formation in living cells [15]–[18]. Our data presented here shows for the first time that hNUDC is able to form a complex with Mpl in the context of living cells (Fig. 1). More importantly, we have identified the N-terminal 159 aa region within hNUDC as a determinant of interaction with Mpl; in contrast, the C-terminal region of hNUDC, containing the evolutionarily conserved NUDC homology domains, is not required for the interaction with Mpl (Fig.1). The results revealed from flow cytometry were consistent with these BiFC studies. Based on the background control (untransfected Cos-1 cells), the Venus signals of hNUDC/Mpl and hNUDC(1-159)/Mpl increased 16.7-fold and 16.2-fold, respectively. In contrast, when probing an interaction between hNUDC(160-331) and Mpl, only a 4.5-fold increase was observed compared to the control. In addition, the corresponding empty vector plasmids produced fluorescent intensities that were 2.5-fold higher than untransfected Cos-1 cells (Fig. 2). Moreover, our data provide support for the use of CFP/YFP FRET pairs in acceptor photobleaching experiments. After photobleaching, green fluorescence intensity is increased in emission spectra for the hNUDC-CFP/Mpl-YFP or hNUDC(1-159)-CFP/Mpl-YFP, but not for hNUDC(160-331)-CFP/Mpl-YFP (Fig. 3). These indicated that both full-length and the N-terminal half of hNUDC were able to undergo interaction with Mpl, but the C-terminal half of hNUDC and Mpl-YFP interaction did not occur.

In addition to allowing quantification of the relative efficiency of interaction between hNUDC and Mpl, BiFC also allows the steady-state localization of the complex. Our confocal microscopy data demonstrated that a hNUDC/Mpl BiFC localization pattern tested herein showed a punctate distribution consistent with potential vesicular localization. Co-transfection of hNUDC-VC/Mpl-VN with plasmid containing ER, Golgi or membrane revealed considerable overlap between the BiFC signal and these markers. These results are in accordance with previous studies that identified the same localization for hNUDC/Mpl in different cell lines [10], [13]. Most notably, the subcellular localization of hNUDC(1-159)/Mpl complex is similar to that of hNUDC/Mpl (Fig. 4). It remains possible that the organization of this complex around the 1-159 aa domain of hNUDC may be essential for interaction with Mpl within cells. Although we did not specifically study the translocation of hNUDC(1-159) in the absence of Mpl, in view of this study, we acknowledge the possibility that the regulation of hNUDC/Mpl mobilization, trafficking, and/or secretion relies on high affinity between N-terminal NUDC and Mpl.

Nonetheless, for all transfection tested with hNUDC/Mpl and hNUDC(1-159)/Mpl BiFC constructs, a large portion of the ER-associated complexes appeared predominantly in the perinuclear region (Fig. 4). Although we do not know exactly how the binding of hNUDC to Mpl induces its retention in the ER, one possibility that the BiFC technique was only able to capture a rapid transient interaction, and the binding between VC and VN fragments in BiFC assays makes the formed complex permanent once they are associated. In this case, the ER retention could not be relieved and allowed transport of hNUDC and Mpl to the plasma membrane. Mpl is a member of the cytokine receptor superfamily [23]. To be fully functional, Mpl need to be delivered to the plasma membrane in a ligand-responsive and signaling-competent form for the regulation of megakaryocyte differentiation and platelet formation [24]. The synthesis and maturation of Mpl may require complex combinations of processes, including protein folding, posttranslational modifications, and transport through distinct cellular compartments. We pursue the hypothesis that retention of misfolded/unassembled Mpl in the perinuclear region by hNUDC may, therefore, represent an important step in the quality control mechanism, ensuring that only properly folded receptors are transported to Golgi and the plasma membrane.

Our results also indicated that overexpression of hNUDC via adenovirus-mediated gene transfer into a megakaryocyte cell line, which naturally expression of Mpl, increased the secretion of hNUDC. Using adenoviral vectors expressing shRNA we have shown that depletion of Mpl dramatically reduced secretion of hNUDC, suggesting that regulation of hNUDC secretion relies on interactions with Mpl. This result is in agreement with previous study in NIH 3T3 cells which suggest that hNUDC secretion is Mpl-dependent, since hNUDC fails to continue its way through the secretory pathway in the absence of Mpl [13]. Although a requirement for Mpl in hNUDC secretion has been established using immunofluorescence and ELISA assays in both Dami and NIH 3T3 cells, the exact molecular role of this protein in synchronized trafficking remains unknown. It is possible that the hNUDC/Mpl complex is transported from the ER to the late Golgi and subsequently to the cell surface in the conventional secretory pathway. We also demonstrated for the first time that the secretion of the N-terminal half of hNUDC is dependent of Mpl. That Mpl governs the coordinated release of hNUDC or hNUDC(1-159) from transfected Dami cells is of fundamental interest and possibly provides a source of extracellular hNUDC for an additional role during the cell proliferation and differentiation. In contrast to hNUDC-VC and hNUDC(1-159)-VC, hNUDC(160-331)-VC failed to secrete when it expressed in Dami cells. It is possible that the N- and C-terminal hNUDC domains could be driven by totally different mechanisms, but this remains to be proven. This hypothesis was highlighted by the fact that the C-terminal hNUDC fused with CFP was diffusively distributed throughout the cytosol independently of Mpl and it did not co-localize with either ER or Golgi (unpublished data).

Previous studies have identified that hNUDC residues 100-238 efficiently bind to Mpl in a yeast two-hybrid system [12]. To investigate whether the same domain could also bind Mpl *in vivo*, the central 100-238 residues of hNUDC were truncated and fused to VC to create phNUDC(ΔN100-283)-VC in an identical manner to the full-length protein. Flow cytometry indicated that cells transfected with phNUDC(ΔN100-283)-VC/Mpl-VN only produced about 15% of the Venus-positive cells that the phNUDC-VC/Mpl-VN combination gave rise to (data not shown), indicating that the same domain in hNUDC is interacting with Mpl *in vivo*. However, it is worthwhile to mention that the truncation of 100–280 residues may not truly reflect its native interaction in the context of the hNUDC-Mpl BiFC complex. It could be possible that the truncation of a central domain triggers conformational changes in the hNUDC protein. Recombinant hNUDC(1-159) and hNUDC(100-238) overlap by 59 aa, which contains an acidic box, DAEEEEDEEDEKDK, that is reminiscent of a repeated sequence found in an NLS-binding protein, Nopp140, which appears to be responsible for conveying other proteins from the cytoplasm to the nucleus [25]. An immediate question that arises from this finding is whether this segment contains sufficient information for association with Mpl. Further amino acid mutagenesis will be important to determine the roles of this conserved site of hNUDC with regards to Mpl interaction.

Previous studies have identified that LIS-1, a homologous protein associated with a fungal nuclear migration protein (NUDE) [26], is also interacting with hNUDC [27]–[29]. In view of previous work and this study, we propose a model in which hNUDC has two independent roles with two functional domains: the N-terminus is preferably bound to Mpl, whereas the C-terminus may interact with other proteins, such as the LIS1-1/dynein motor complex [30]–[32]. However, the conclusions we can draw from these results are limited, and the existence of a ternary complex of hNUDC, Mpl and LIS-1 requires additional clarification. Determining whether all the observed complexes of hNUDC/Mpl and hNUDC/LIS-1 coexist in one cell at the same time remains an important area of future investigation.
